# Reversion to normoglycemia with tirzepatide vs semaglutide in participants with obesity and prediabetes: a post hoc analysis of SURMOUNT-5

**DOI:** 10.1007/s40618-026-02895-3

**Published:** 2026-04-20

**Authors:** Rodolfo J. Galindo, Louis J. Aronne, Deborah B. Horn, Alexander D. Miras, Julia P. Dunn, Dachuang Cao, Clare J. Lee, Chrisanthi A. Karanikas, Robert J. Heine, Beverly L. Falcon, Georgios K. Dimitriadis

**Affiliations:** 1 Division of Endocrinology, Diabetes and Metabolism, Department of Medicine, University of Miami Miller School of Medicine, Miami, FL USA; 2 Division of Endocrinology, Diabetes and Metabolism, Weill Cornell Medicine, Comprehensive Weight Control Center, New York, NY USA; 3 Center for Obesity Medicine and Metabolic Performance, Departments of Internal Medicine and Surgery, University of Texas McGovern Medical School, Houston, TX USA; 4 School of Medicine, Ulster University, Derry, UK; 5 Department of Metabolism, Digestion and Reproduction, Imperial College London, London, SW7 2AZ UK; 6 Eli Lilly and Company, Indianapolis, IN USA; 7 Eli Lilly and Company Ltd., Basingstoke, UK

**Keywords:** Tirzepatide, Semaglutide, Obesity, Prediabetes, SURMOUNT-5, Clinical trial, Post hoc analysis

## Abstract

**Background:**

Prevention of progression into type 2 diabetes (T2D) in patients with prediabetes is a key goal of obesity management. In SURMOUNT-5, once weekly tirzepatide at the maximum tolerated dose (MTD 10 mg or 15 mg) compared with semaglutide (MTD 1.7 mg or 2.4 mg) resulted in significantly greater body weight reduction in adults living with obesity without T2D.

**Purpose:**

Assess changes in glycemia outcomes with tirzepatide (MTD 10 mg or 15 mg) compared with semaglutide (MTD 1.7 mg or 2.4 mg) in participants with obesity and prediabetes from SURMOUNT-5.

**Methods:**

Participants (N = 425) with baseline prediabetes, defined as having ≥ 1 fasting lab-based value of either FSG 100–125 mg/dL or HbA1c 5.7–6.4%, were included in this analysis. Change from baseline in HbA1c, proportion of participants achieving normoglycemia (i.e., HbA1c < 5.7% and FSG < 100 mg/dL), percent change from baseline in body weight, fasting insulin, estimates of insulin sensitivity (HOMA2-IR), and proportion of participants achieving body weight reduction thresholds (≥ 10% to ≥ 30%) at Week 72 were assessed using MMRM or logistic regression for categorical measures using the efficacy analysis set.

**Results:**

Mean baseline age was 47 years, 63% were female, BMI was 40 kg/m^2^, and HbA1c was 5.86%. At Week 72, mean HbA1c reduction was significantly greater with tirzepatide vs semaglutide (-0.60% vs -0.48%; estimated treatment difference [ETD; 95% CI] -0.12% [−0.18, −0.06]; p < 0.001). A greater proportion of tirzepatide-treated participants reverted to normoglycemia (89.9%) vs semaglutide (76.2%). Mean percent body weight reduction was significantly greater with tirzepatide (−21.5% vs -14.5%; ETD −7.1% [−9.1, −5.0]; p < 0.001). Greater improvements in fasting insulin and HOMA2-IR were observed with tirzepatide vs semaglutide (p < 0.001).

**Conclusion:**

In this post hoc analysis of SURMOUNT-5, a greater proportion of tirzepatide-treated participants with obesity and prediabetes at baseline reverted to normoglycemia compared with semaglutide. Greater improvements in glycemia, estimates of insulin sensitivity and body weight were also observed with tirzepatide.

**Supplementary Information:**

The online version contains supplementary material available at 10.1007/s40618-026-02895-3.

## Introduction

Obesity and overweight are the most common modifiable etiologic risk factors for type 2 diabetes (T2D). The global prevalence of obesity is expected to grow to 1.13 billion by 2030 [1]. Furthermore, the prevalence of prediabetes in adults is also rising [2]. In the U.S., it is estimated that 84 million adults have prediabetes, of whom 85% have overweight or obesity [3]. In the National Diabetes Prevention Program, a well-structured randomized controlled trial, intensive lifestyle interventions demonstrated a significant reduction in the incidence of T2D by 58% in a high-risk population with prediabetes [4, 5]. However, several studies have failed to translate the long-term effect, adherence, and sustainability of these intensive lifestyle programs into real-world settings. It is therefore important to address these interconnected and growing epidemics with effective, safe, and well-tolerated interventions. Tirzepatide, a long-acting glucose-dependent insulinotropic polypeptide (GIP) and glucagon-like peptide-1 (GLP-1) receptor agonist [6], and semaglutide, a long-acting GLP-1 receptor agonist [7], are the first two approved highly effective obesity and diabetes management medications [8].

In the placebo-controlled Semaglutide Treatment Effect in People with Obesity (STEP) 10 clinical trial for participants with obesity and prediabetes, once weekly semaglutide 2.4 mg provided superior reduction in body weight compared with placebo (14% vs 3%) and a significantly greater proportion of participants achieving normoglycemia, defined as a glycated hemoglobin (HbA1c) level < 5.7% (38.8 mmol/mol) (81% vs 14%, respectively. [9] Furthermore, in the phase 3 randomized, placebo-controlled 3-year SURMOUNT-1 trial for people with obesity and prediabetes, tirzepatide compared with placebo resulted in substantial and sustained weight reduction (15–23% vs 2%), significant reduction in HbA1c (0.50–0.65% vs 0.14%), as well as a 94% reduction in risk of progression to T2D [10]. SURMOUNT-5 is the first head-to-head trial of highly effective obesity management medications tirzepatide and semaglutide in participants with obesity with and without prediabetes at baseline [11]. Following 72 weeks of treatment, tirzepatide at the maximum tolerated dose (MTD, 10 mg or 15 mg) resulted in superior reductions in body weight compared with semaglutide MTD (1.7 mg or 2.4 mg) (22% vs 15%) [11].

In this post hoc analysis of SURMOUNT-5, we assessed the proportion of participants achieving normoglycemia, changes in glycemic control and related estimates of insulin resistance and insulin sensitivity, and changes in body weight with tirzepatide vs semaglutide among participants with obesity and prediabetes at baseline.

## Research design and methods

### Trial design and participants

Study design, full inclusion and exclusion criteria, and primary results are published [11]. Briefly, SURMOUNT-5 was a phase 3b multicenter, randomized, parallel-arm, open-label, active-controlled, 72-week trial that included adults aged 18 years or older, with a body mass index (BMI) ≥ 30 kg/m^2^ or a BMI ≥ 27 kg/m^2^ with at least one obesity-related complication (ORC, i.e., hypertension, dyslipidemia, obstructive sleep apnea, or cardiovascular disease), and relatively stable weight, not more than 5 kg change in 3 months before screening. Key exclusion criteria included a diabetes diagnosis or screening HbA1c or fasting serum glucose (FSG) supportive of T2D [12], past or planned surgical treatment for obesity, and treatment with a medication for body weight reduction or a GLP-1 receptor agonist within 3 months prior to screening. Participants were randomized (1:1) to once weekly tirzepatide MTD (10 mg or 15 mg) or semaglutide MTD (1.7 mg or 2.4 mg) as an adjunct to a reduced calorie diet and increased physical activity. Participants were stratified by baseline presence of prediabetes based on screening HbA1c and FSG [12], sex (female/male) and baseline BMI (< 35, ≥ 35 kg/m^2^). Dose escalation was consistent with both medications’ approved labels. Participants were intended to reach the maximum dose, but the lower dose was allowed in case of persistent gastrointestinal intolerance.

The SURMOUNT-5 trial (clinicaltrials.gov identifier NCT05822830) was conducted in accordance with the International Conference on Harmonisation Guidelines for Good Clinical Practice and the Declaration of Helsinki. All participants provided signed informed consent, and protocols were approved by local ethical review boards.

### Definitions of prediabetes

In the SURMOUNT-5 trial, prediabetes was defined as having at least one fasting lab-based value of either FSG 100–125 mg/dL (6–7 mmol/L) or HbA1c 5.7–6.4% (39–46 mmol/mol) at screening based on the American Diabetes Association [12]. In the current analysis, prediabetes status was also assessed as defined by the World Health Organization/International Expert Committee (WHO/IEC), which includes a FSG level 110–125 mg/dL (6–7 mmol/L) and/or HbA1c 6.0–6.4% (42–46 mmol/mol) [13, 14]. The criterion of a 2-h plasma glucose level of 140–199 mg/dL (8–11 mmol/L) during 75 g oral glucose-tolerance test (OGTT) was not applicable as dynamic assessments were not performed in this study. Results for the WHO/IEC-defined prediabetes subgroup are provided in the Supplemental Appendix.

### Endpoint

Change in glycemia was assessed by changes from baseline in HbA1c and FSG, and percent change from baseline in fasting insulin and estimates of steady-state beta-cell function and insulin sensitivity using the homeostasis model assessment computed with insulin (i.e., HOMA2-IR, HOMA2-S and HOMA2-B) at Week 72. The proportion of participants achieving HbA1c targets of < 6.0% and < 5.7% at Week 72 were assessed. Furthermore, normoglycemia, defined as an HbA1c < 5.7% and FSG < 100 mg/dL, was assessed in the study-defined prediabetes subgroup and normoglycemia defined as an HbA1c < 6.0% and FSG < 110 mg/dL, was assessed in the WHO/IEC prediabetes subgroup. Percent change from baseline in body weight, change from baseline in waist circumference and waist-to-height ratio (WHtR), and the proportion of participants achieving body weight reduction thresholds (≥ 10%, ≥ 15%, ≥ 20%, ≥ 25%, and ≥ 30%) were also assessed at Week 72. Additional analysis was performed to estimate the association of body weight reduction on changes in HOMA2-IR (computed with insulin) with tirzepatide. Laboratory measurements were collected via a central laboratory [11]. Body weight by glycemic status was a prespecified analysis while all other endpoints were post hoc [11].

### Statistical analyses

The efficacy analysis set (i.e., randomized and treated participants, excluding off-treatment data) using the efficacy estimand was used for this analysis. Change from baseline at Week 72 in HbA1c, percent change from baseline at Week 72 in body weight, fasting insulin, and HOMA2 parameters (i.e., HOMA2-IR, HOMA2-S and HOMA2-B) were assessed using mixed model for repeated measures with treatment group, time, treatment-by-time interaction, prediabetes status at randomization, sex, and BMI category as fixed effects, and baseline value of the response variable as a covariate and reported as least squares means and standard errors with fasting insulin and HOMA2 parameters reported as overall baseline geometric mean, estimate (standard error) actual values and percent change from baseline at Week 72 using log transformation. Proportions of participants achieving body weight reduction thresholds (≥ 10% to ≥ 30%) were summarized using logistic regression based on imputed data. Missing data at Week 72 due to early treatment discontinuation, initiation of prohibited medications or bariatric surgery or other weight loss procedures were assumed to be missing at random. In this case, multiple imputation was based on data from participants in the same treatment arm. Missing data were imputed 100 times. Statistical inference for endpoint uses Rubin’s Rule to combine analyses of imputed datasets. Additional analysis was done to determine the proportion of the estimated treatment difference in HOMA2-IR with tirzepatide versus semaglutide that was attributable to an indirect association with body weight reduction. The model for estimation of weight loss-dependent and weight loss-independent effects on HOMA2-IR at Week 72 included interaction between treatment and body weight change, baseline HOMA2-IR, sex, and baseline BMI group (< 35, ≥ 35 kg/m^2^).

## Results

### Baseline demographic and clinical characteristics

In SURMOUNT-5, 425 participants had study-defined prediabetes at baseline (tirzepatide N = 215, semaglutide N = 210). The baseline demographics and clinical characteristics among these participants were well-balanced across the treatment groups (Table 1). Overall, mean baseline age was 47.3 years, duration of obesity was 16.5 years, body weight was 115.8 kg, BMI was 40.4 kg/m^2^, WHtR was 0.71, HbA1c was 5.86%, FSG was 98.8 mg/dL and fasting insulin was 163.5 pmol/L. Additionally, 48% of participants had hypertension and 37% had dyslipidemia at baseline defined by medical history questioning. Most participants were female (63%) and of White race (73%).

**Table 1:** Baseine demolgraphics and clinical characteristics in participants with study-defined prediabetes from SURMOUNT-5

Parameter	Tirzepatide MTD (10 or 15 mg) N = 215	Semaglutide MTD (1.7 or 2.4 mg) N = 210	Total population N = 425
**Age, years**	47.6 ± 12.9	47.0 ± 12.7	47.3 ± 12.8
**Sex, n (%)**			
Male	81 (37.7)	77 (36.7)	158 (37.2)
Female	134 (62.3)	133 (63.3)	267 (62.8)
**Race, n (%)**			
American Indian or Alaska Native	4 (1.9)	0	4 (0.9)
Asian	8 (3.7)	6 (2.9)	14 (3.3)
Black or African American	51 (23.7)	43 (20.5)	94 (22.1)
Multiple	3 (1.4)	2 (1.0)	5 (1.2)
White	149 (69.3)	159 (75.7)	308 (72.5)
**Ethnicity, n (%)**			
Hispanic or Latino	50 (23.3)	58 (27.6)	108 (25.4)
Not Hispanic or Latino	165 (76.7)	152 (72.4)	317 (74.6)
**HbA1c, %**	5.83 ± 0.28	5.88 ± 0.29	5.86 ± 0.28
**FSG, mg/dL**	98.8 ± 10.56	98.9 ± 10.20	98.8 ± 10.37
**Fasting insulin, pmol/L**	157.8 ± 113.1	169.4 ± 127.1	163.5 ± 120.2
**Duration of obesity, years**	17.7 ± 11.72	15.2 ± 11.41	16.5 ± 11.62
**Body weight, kg**	115.5 ± 24.82	116.1 ± 27.42	115.8 ± 26.11
**BMI, kg/m**^**2**^	40.4 ± 8.00	40.3 ± 8.13	40.4 ± 8.05
< 35 kg/m^2^, n (%)	54 (25.1)	54 (25.7)	108 (25.4)
≥ 35 kg/m^2^, n (%)	161 (74.9)	156 (74.3)	317 (74.6)
**Waist circumference, cm**	120.0 ± 16.09	120.8 ± 17.66	120.4 ± 16.87
**Waist-to-height ratio**	0.71 ± 0.09	0.71 ± 0.10	0.71 ± 0.09
**Systolic blood pressure, mmHg**	127.6 ± 13.48	128.1 ± 12.03	127.9 ± 12.77
**Diastolic blood pressure, mmHg**	81.6 ± 8.20	81.9 ± 7.79	81.7 ± 7.99
**Pulse rate, bpm**	72.1 ± 9.58	72.2 ± 9.90	72.2 ± 9.73
**eGFR, mL/min/1.73m**^**2**^	102.3 ± 17.21	103.5 ± 17.08	102.9 ± 17.13
**Hypertension, n (%)**	109 (50.7)	96 (45.7)	205 (48.2)
**Dyslipidemia, n (%)**	80 (37.2)	78 (37.1)	158 (37.2)
**Prediabetes, n (%)**			
HbA1c ≥ 5.7%	177 (82.3)	182 (86.7)	359 (84.5)
FSG ≥ 100 mg/dL	109 (50.9)	100 (47.6)	209 (49.3)
HbA1c ≥ 5.7% and FSG ≥ 100 mg/dL	71 (33.2)	72 (34.3)	143 (33.7)
**Number of ORCs, including prediabetes, n (%)**			
1	48 (22.3)	44 (21.0)	92 (21.6)
2	65 (30.2)	77 (36.7)	142 (33.4)
≥ 3	102 (47.4)	89 (42.4)	191 (44.9)

Data are mean ± SD, unless otherwise indicated, in participants with prediabetes at randomization from the modified intent-to-treat population. Note: Prediabetes defined by HbA1c ≥ 5.7% only and FSG ≥ 100 mg/dL only includes participants meeting both criteria. Abbreviations: BMI = body mass index; eGFR = estimated glomerular filtration rate (from creatinine adjusted for body surface area); FSG = fasting serum glucose; HbA1c = glycated hemoglobin; MTD = maximum tolerated dose; ORCs = obesity-related complications; SD = standard deviation

Baseline demographics and clinical characteristics of participants with WHO/IEC-defined prediabetes are presented in Table S1.

### Changes in glycemic parameters

**Fig. 1:**
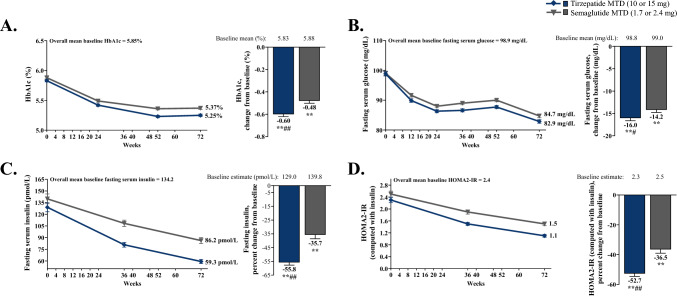
Change in glycemic parameters in participants with study-defined prediabetes from SURMOUNT-5**.** Data are LSM (SE) actual values over time and change from baseline at Week 72 from the efficacy analysis set. For fasting insulin and HOMA2 parameters, values are overall baseline geometric mean, estimate (SE) actual values and percent change from baseline at Week 72 using log transformation. **p < 0.001 change from baseline; #p < 0.05 and ##p < 0.001 vs semaglutide. (**A**) HbA1c (%) over time and change from baseline at Week 72. (**B**) Fasting serum glucose (mg/dL) over time and change from baseline at Week 72. (**C**) Fasting insulin (pmol/L) over time and change from baseline at Week 72. (**D**) HOMA2-IR (computed with insulin) over time and percent from baseline at Week 72. Abbreviations: HOMA2-IR = homeostatic model assessment for insulin resistance; LSM = least squares mean; MTD = maximum tolerated dose; SE = standard error

**Fig. 2:**
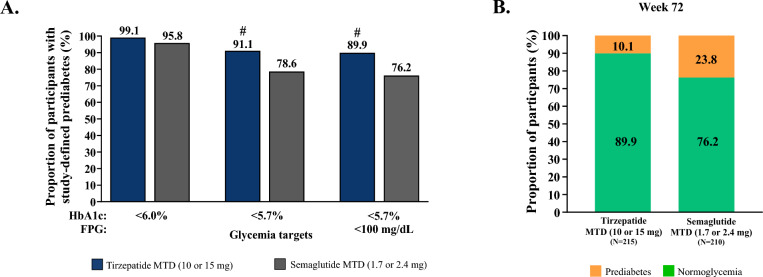
Proportion of participants with study-defined prediabetes achieving glycemia targets at Week 72 from SURMOUNT-5**.** Data are percentage of participants achieving HbA1c targets based on imputed data and proportion of participants with normoglycemia (i.e., HbA1c < 5.7% and FSG < 100 mg/dL) or prediabetes at Week 72 from the efficacy analysis set. #p < 0.05 and ##p < 0.001 vs semaglutide. Note: Missing data at Week 72 due to early treatment discontinuation, initiation of prohibited medications or bariatric surgery or other weight loss procedures were assumed to be missing at random. In this case, multiple imputation was based on data from participants in the same treatment arm. Missing data were imputed 100 times. Statistical inference for endpoint uses Rubin’s Rule to combine analyses of imputed datasets. (**A**) Proportion of participants with study-defined prediabetes achieving HbA1c targets at Week 72. (**B**) Glycemic status at Week 72. Abbreviations: MTD = maximum tolerated dose

Among participants with study-defined prediabetes, mean (95% confidence interval) HbA1c was significantly reduced from baseline in the tirzepatide group by 0.60% (−0.64, −0.56) and in the semaglutide group by 0.48% (−0.52, −0.44) at Week 72 (p < 0.001, both groups) (Fig. 1A). Mean HbA1c reductions were significantly greater with tirzepatide vs semaglutide (estimated treatment difference [95% confidence interval] −0.12% [−0.18, −0.06]; p < 0.001). At Week 72, a significantly greater proportion of tirzepatide- vs semaglutide-treated participants achieved HbA1c < 5.7% (91.1% vs 78.6%) and HbA1c < 5.7% and FSG < 100 mg/dL (89.9% vs 76.2%) (Fig. 2A and B). The risk difference (95% confidence interval) for the proportion of participants achieving an HbA1c target < 5.7% with tirzepatide vs semaglutide was 10.5% (3.6, 17.5); p = 0.003 and for the proportion of participants achieving an HbA1c target < 5.7% and FSG < 100 mg/dL with tirzepatide vs semaglutide was 11.4% (4.2, 18.6); p = 0.002.

Significant reductions from baseline in mean (95% confidence interval) FSG were also observed with tirzepatide by 16.0 mg/dL (−17.18, −14.89) and with semaglutide by 14.2 mg/dL (−15.37, −13.01) (p < 0.001, both groups) and were significantly greater with tirzepatide (−1.85 mg/dL [−3.49, −0.20]; p = 0.028) (Fig. 1B). Fasting insulin significantly reduced from baseline by 55.8% with tirzepatide and by 35.7% with semaglutide (p < 0.001, both groups), and reductions were significantly greater with tirzepatide (−31.2% [−39.2, −22.2]; p < 0.001) (Fig. 1C).

Overall, greater improvements in HOMA2-IR (computed with insulin) were observed with tirzepatide compared with semaglutide. At Week 72, HOMA2-IR significantly decreased from baseline with tirzepatide by 52.7% and with semaglutide by 36.5% (p < 0.001, both treatment groups) and this reduction was significantly greater with tirzepatide (−25.4% [−33.3, −16.6]; p < 0.001) (Fig. 1D). Change in additional HOMA2 parameters (i.e., HOMA2-S and HOMA2-B) are presented in Figure S1.

Overall, similar trends in glycemic parameters were observed among participants with WHO/IEC-defined prediabetes (Figures S2 and S3A).

### Changes in body weight-related parameters

**Fig. 3:**
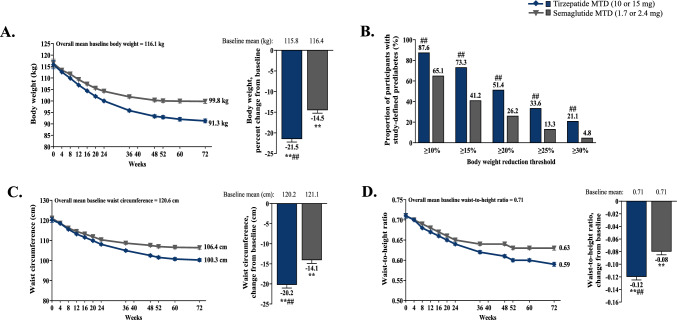
Change in body weight-related parameters and proportion of participants achieving body weight reduction thresholds at Week 72 in participants with study-defined prediabetes from SURMOUNT-5. Data are LSM (SE) actual values and change or percent change from baseline at Week 72 and percentage of participants based on imputed data from the efficacy analysis set. **p < 0.001 change from baseline; #p < 0.05 and ##p < 0.001 vs semaglutide. (A) Body weight (kg) over time and percent change from baseline at Week 72 in participants with study-defined prediabetes. (B). Proportion of participants with study-defined prediabetes achieving body weight reduction thresholds at Week 72. Note: Missing data at Week 72 due to early treatment discontinuation, initiation of prohibited medications or bariatric surgery or other weight loss procedures were assumed to be missing at random. In this case, multiple imputation was based on data from participants in the same treatment arm. Missing data were imputed 100 times. Statistical inference for endpoint uses Rubin’s Rule to combine analyses of imputed datasets. (C) Waist circumference (cm) over time and change from baseline at Week 72 in participants with study-defined prediabetes. (D) Waist-to-height ratio over time and change from baseline at Week 72 in participants with study-defined prediabetes. Abbreviations: LSM = least squares mean; MTD = maximum tolerated dose; SE = standard error

Among participants with study-defined prediabetes, mean (95% confidence interval) percent body weight reduction observed with tirzepatide was 21.5% (−22.94, −20.07) and with semaglutide was 14.5% (−15.91, −12.99) at Week 72 (p < 0.001, both groups) (Fig. 3A). The reduction in body weight was significantly greater with tirzepatide compared with semaglutide (−7.1% [−9.1, −5.0]; p < 0.001). A significantly greater proportion of participants achieved all body weight reduction thresholds with tirzepatide vs semaglutide, with 87.6% vs 65.1% achieving body weight reductions ≥ 10%, 73.3% vs 41.2% achieving body weight reductions ≥ 15%, 51.4% vs 26.2% achieving body weight reductions ≥ 20%, 33.6% vs 13.3% achieving body weight reductions ≥ 25%, and 21.1% vs 4.8% achieving body weight reductions ≥ 30%, respectively (all p < 0.001 compared with semaglutide) (Fig. 3B). The risk differences (95% confidence intervals) for tirzepatide vs semaglutide were 22.7% (14.3, 31.0), 32.2% (23.2, 41.3), 25.4% (16.2, 34.6), 20.6% (12.7, 28.5), 16.8% (10.7, 23.0), for each respective threshold (all p < 0.001 compared with semaglutide). Additionally, significant reductions from baseline in waist circumference were observed with tirzepatide (−20.2 cm [−21.80, −18.68]) and semaglutide (−14.1 cm [−15.68, −12.51]) (p < 0.001, both groups) and were significantly greater with tirzepatide (−6.2 cm [−8.4, −3.9]; p < 0.001) (Fig. 3C), as well as significant reductions in WHtR (−0.12 vs −0.08; ETD: −0.04 [−0.05, −0.02]; p < 0.001) (Fig. 3D).

Overall, similar improvements in body weight parameters were observed among participants with WHO/IEC-defined prediabetes (Figure S3B and Figure S4).

### Association of body weight reduction and changes in HOMA2-IR

The relationship between body weight reduction and changes in HOMA2 parameters was assessed among the subgroups of participants with study-defined prediabetes treated with tirzepatide or semaglutide. At Week 72, reductions in body weight were associated with decreases in insulin resistance, as indicated by HOMA2-IR, with both tirzepatide and semaglutide treatment. These associations were statistically significant, with correlation coefficients of r = 0.33300 (p < 0.0001) in the tirzepatide treatment group and r = 0.30072 (p = 0.0001) in the semaglutide-treatment group (Figure S5A). The relationship between body weight reduction and changes in HOMA2-S and HOMA2-B are presented in Figure S5B and Figure S5C. Overall, similar improvements in HOMA2 parameters were observed among participants with WHO/IEC-defined prediabetes (Figure S6).

Among participants with study-defined prediabetes, body weight reduction explained 52% (95% confidence interval [30.3, 87.8]) of the estimated treatment difference in HOMA2-IR observed at Week 72 (Table S2).

### Safety profile

In the overall population from SURMOUNT-5, 2 (0.3%) self-reported adverse events of T2D were reported, one in each treatment arm. The overall safety profile among participants with study-defined prediabetes was similar to the overall population from SURMOUNT-5 (Table S3). At least one treatment-emergent adverse event (TEAE) was reported by 79% of tirzepatide-treated participants and 83% of semaglutide-treated participants with study-defined prediabetes. The most commonly reported TEAEs were gastrointestinal-related, including nausea (46% vs 44%), constipation (27% vs 31%), diarrhea (25% vs 25%) and vomiting (15% vs 21%) with tirzepatide compared with semaglutide, respectively.

## Discussion

In this post hoc analysis of SURMOUNT-5, a greater proportion of participants with obesity and prediabetes at baseline reverted to normoglycemia following 72 weeks of tirzepatide treatment as compared with semaglutide; in addition, greater improvements in glycemic parameters, including mean HbA1c, FSG, insulin and HOMA2-IR were associated with tirzepatide. Furthermore, treatment with tirzepatide was associated with greater reductions in mean percent body weight, mean waist circumference and WHtR compared with semaglutide, with a higher proportion of tirzepatide-treated participants achieving all body weight reduction thresholds. Overall, similar body weight-related and anthropometric findings were observed for participants with WHO/IEC-defined prediabetes.

The current differences shown between tirzepatide and semaglutide for improved, albeit modest, glycemic control, and improved body weight-related parameters and anthropometric indices are consistent with those reported in the respective placebo-controlled studies of tirzepatide and semaglutide among individuals with obesity and prediabetes [9, 10]. In the STEP 1, 3 and 4 trials, 84–90% of participants with obesity and prediabetes at baseline reverted to normoglycemia following 68 weeks of treatment with semaglutide 2.4 mg compared with 48–70% of participants treated with placebo (including participants who received semaglutide during a 20-week run-in period) [15]. Similarly, in SURMOUNT-1, 95% of tirzepatide-treated participants who had prediabetes at baseline reverted to normoglycemia, as compared with 62% of placebo-treated participants at Week 72 [16], with benefits shown to be sustained with additional 2-year follow-up [10].

In a post hoc analysis of the SURMOUNT-1 trial, Mari et al., observed improvements in insulin sensitivity and beta-cell function associated with tirzepatide treatment at Week 72 in the overall population, as well as among subgroups of participants with either prediabetes or normoglycemia at baseline [17]. In that study, beta-cell function was assessed using a validated model of insulin secretion during a 75-g OGTT performed at baseline and Week 72. The main beta-cell function parameters were 1) insulin secretion rate (ISR); 2) beta-cell glucose sensitivity (β-GS); 3) ISR at a fixed glucose concentration of 5.4 mmol/L (ISR5.4 mmol/L); 4) total insulin secretion during the OGTT; 5) potentiation of ISR; and 6) beta-cell rate sensitivity (β-RS). Using multivariate regression models, improvements in insulin sensitivity were mostly associated with body weight reduction and only partly attributed to tirzepatide treatment, whereas enhancements in beta-cell function, and in particular with β-GS and ISR5.4 mmol/L, showed an inverse relationship with percentage change in body weight and were mostly associated with tirzepatide treatment [17].

In a recent prespecified analysis of the SELECT trial, participants with overweight or obesity and preexisting cardiovascular disease and prediabetes demonstrated increased reversion to normoglycemia and reduced progression to diabetes following long-term semaglutide treatment compared with placebo, although glycemic control deteriorated slightly over time at a similar rate between both groups [18]. In another analysis, tirzepatide treatment as compared with semaglutide was associated with greater improvement in insulin sensitivity in participants with T2D, with benefits proportional, but not solely attributable, to the observed reductions in body weight and fat mass [19]. Notably, tirzepatide treatment was also associated with greater improvement in insulin sensitivity per unit body weight loss than with semaglutide [19]. Furthermore, in participants with T2D, treatment with the selective long-acting GIP receptor agonist, macupatide, was associated with a larger insulin sensitivity improvement compared with the selective GLP-1 receptor agonist dulaglutide, despite similar body weight reduction, and combination therapy with these agents was associated with additive effects on insulin sensitivity [20]. In the current study, we observed a greater improvement in insulin sensitivity with tirzepatide compared with semaglutide. In this study, the difference in body weight reduction between tirzepatide- and semaglutide-treated groups can only partly explain the improvement in insulin sensitivity, lending support to the observation that tirzepatide was associated with improvements in insulin sensitivity associated with both body weight-lowering and a potential insulin-sensitizing effects of GIP. Furthermore, participants treated with tirzepatide experienced statistically greater improvements in waist circumference and WHtR compared with those treated with semaglutide. Central adiposity has been linked to a higher risk of progression to T2D by several studies, and loss of visceral adipose tissue is critical in the remission of prediabetes and prevention of T2D development. The current findings warrant future mechanistic studies addressing incretin induced changes in the natural history of prediabetes. It is posited that the greater proportion of tirzepatide-treated participants reverting to normoglycemia and greater improvement in insulin sensitivity may be related to GIP receptor activation [19] – as a differentiating factor between the two molecules studied, among other causes.

The concomitant improvement in insulin sensitivity and beta-cell function, which were associated with normalization of glucose levels in people with prediabetes, suggests that tirzepatide treatment may delay and potentially halt progression to T2D, by inducing so-called “beta-cell rest” and ultimately, modifying the natural history of prediabetes/T2D. In the Diabetes Prevention Study, metformin use led to a 31% reduction in progression to diabetes over a mean follow-up of 2.8 years [4]. Similarly, in the ORIGIN trial, insulin decreased progression by 28% after a median of 6.2 years [21]. Additionally, in the Da Qing Diabetes Prevention Study, a randomized controlled trial in patients with impaired glucose tolerance at baseline, lifestyle interventions in patients with impaired glucose tolerance at baseline reduced the incidence of cardiovascular and all-cause mortality and diabetes incidence, as well as the incidence of diabetes-related microvascular complications (specifically retinopathy) [22, 23]. Evidence supports that prediabetes is not simply a state of pre-diagnosis as both microvascular and macrovascular complications occur in patients with prediabetes even without the transition to diabetes mellitus [24]. Metabolic surgery also lowers microvascular risk in patients with prediabetes, though the underlying mechanism remains unclear [25]. Results from the current post hoc analysis are supported by earlier finding in individuals with obesity and prediabetes [10, 26].

This study had some limitations. First, this was a post hoc analysis and exploratory in nature. Furthermore, the open-label nature of the SURMOUNT-5 trial [11] may have introduced potential differences in adherence and behavioral co-interventions; however, findings were generally consistent with those from previous blinded trials of tirzepatide [10, 16]. This study provided static model-based estimates of insulin resistance, and beta-cell function was not dynamically assessed. While these estimates correspond well, they are not inherently equivalent to non-steady state measures of beta-cell function and insulin sensitivity obtained from stimulatory models. Although race and ethnicity among U.S. participants was representative of U.S. demographics, 70% of participants were White.

In conclusion, in this post hoc analysis of the SURMOUNT-5 trial, a significantly greater proportion of participants with obesity and prediabetes at baseline reverted to normoglycemia following 72 weeks of treatment with tirzepatide as compared with semaglutide, with clinically significant reductions in body weight and waist circumference.

## Duality of interest

**RJG** reports receiving grants from Boehringer Ingelheim, Dexcom, and Novo Nordisk, and receiving consultant/advisory fees from Abbott Diabetes, AstraZeneca, Bayer, Boehringer Ingelheim, Dexcom, Eli Lilly and Company, Medtronic, and Novo Nordisk. **LJA** reports receiving grants or personal fees from Altimmune, AstraZeneca, Boehringer Ingelheim, Eli Lilly and Company, ERX, Gelesis, Intellihealth, Jamieson Wellness, Janssen, Novo Nordisk, Optum, Pfizer, Senda Biosciences, and Versanis and being a shareholder of ERX Pharmaceuticals, Gelesis, Intellihealth, and Jamieson Wellness. **DBH** has acted as a consultant, advisory board member, and speaker for Eli Lilly and Company and Novo Nordisk; has acted as an advisory board member for Amgen AstraZeneca, Boehringer Ingelheim, Zealand; and has received institutional research funding from Eli Lilly and Company, KVK Tech, Novo Nordisk, and Weight Watchers. **ADM** has received research funding from the European Union, Medical Research Council (MRC), National Institute for Health and Care Research (NIHR), HSC R&D division, Jon Moulton Charitable Foundation, Anabio, Fractyl, Boehringer Ingelheim, Eli Lilly and Company, Gila, Randox, and Novo Nordisk. **ADM** has received honoraria for lectures and presentations from Novo Nordisk, AstraZeneca, Currax Pharmaceuticals, Boehringer Ingelheim, Screen Health, GI Dynamics, Algorithm, Eli Lilly and Company, Ethicon, and Medtronic. **ADM** is a shareholder in the Beyond BMI clinic, which provides clinical obesity care. **JPD**, **DC**, **CJL**, **BLF**, **CAK**, **RJH** and **GKD** are employees and shareholders of Eli Lilly and Company.

## Supplementary Information

Below is the link to the electronic supplementary material.

Supplementary file4 (DOCX 875 KB)

## Data Availability

Lilly provides access to all individual participant data collected during the trial, after anonymization, with the exception of pharmacokinetic or genetic data. Data are available to request 6 months after the indication studied has been approved in the US and EU and after primary publication acceptance, whichever is later. No expiration date of data requests is currently set once data are made available. Access is provided after a proposal has been approved by an independent review committee identified for this purpose and after receipt of a signed data sharing agreement. Data and documents, including the study protocol, statistical analysis plan, clinical study report, blank or annotated case report forms, will be provided in a secure data sharing environment. For details on submitting a request, see the instructions provided at www.vivli.org.
