# Supplementary material for: Reversion to normoglycemia with tirzepatide vs semaglutide in participants with obesity and prediabetes: a post hoc analysis of SURMOUNT-5
Source: J Endocrinol Invest. 2026 Apr 20;49(7):1611–20. doi: 10.1007/s40618-026-02895-3 (PMC13451520; doi:10.1007/s40618-026-02895-3)
Supplement: Supplementary file 1 [file 40618_2026_2895_MOESM4_ESM.docx]

**SUPPLEMENTAL APPENDIX**

**Reversion to normoglycemia with tirzepatide vs semaglutide in participants with obesity and prediabetes: A post hoc analysis of SURMOUNT-5**

Rodolfo J. Galindo, Louis J. Aronne, Deborah B. Horn, Alexander D. Miras, Julia P. Dunn, Dachuang Cao, Clare J. Lee, Chrisanthi A. Karanikas, Robert J. Heine, Beverly L. Falcon, Georgios K. Dimitriadis

**This supplement provides data on: Tirzepatide compared with semaglutide in participants with obesity and prediabetes: A post hoc analysis of SURMOUNT-5**

**CONTENTS**

**SUPPLEMENTAL TABLES**

Table S1. Baseline demographics and clinical characteristics in participants with WHO/IEC-defined prediabetes from SURMOUNT-5.....………………………..………………………...……………………2

Table S2. Body weight reduction-associated and -unassociated changes on HOMA2-IR (computed with insulin) in participants with prediabetes from SURMOUNT-5…………………..………………….……...3

Table S3. Safety profile……………………………………………………………………………………..4

**SUPPLEMENTAL FIGURES**

Figure S1. Change in additional HOMA2 parameters in participants with study-defined prediabetes from SURMOUNT-5……………………………………………………………………………………………..5

Figure S2. Change in glycemic parameters in participants with WHO/IEC-defined prediabetes from SURMOUNT-5…………….………….....………………………………………………..……..…………6

Figure S3. Proportion of participants with WHO/IEC-defined prediabetes achieving glycemia targets and body weight reduction thresholds at Week 72 from SURMOUNT-5……...………………………………..7

Figure S4. Change in body weight-related parameters in participants with WHO/IEC-defined prediabetes from SURMOUNT-5…………………...………………...…………………………...……………………8

Figure S5. Relationship between reductions in body weight (kg) and HOMA2 parameters at Week 72 in participants with study-defined prediabetes...………………………………………………………....……9

Figure S6. Relationship between reductions in body weight (kg) and HOMA2 parameters at Week 72 in participants with WHO/IEC-defined prediabetes…………………………………………………….....…10

**Table S1. Baseline demographics and clinical characteristics in participants with WHO/IEC-defined prediabetes from SURMOUNT-5**

| **Parameter** | **Tirzepatide MTD (10 or 15 mg)**  **N=90** | **Semaglutide MTD (1.7 or 2.4 mg)**  **N=96** | **Total Population N=186** |
| --- | --- | --- | --- |
| **Age, years** | 49.4 ± 13.8 | 49.5 ± 13.0 | 49.4 ± 13.4 |
| **Sex, n (%)** |  |  |  |
| Male | 38 (42.2) | 38 (39.6) | 76 (40.9) |
| Female | 52 (57.8) | 58 (60.4) | 110 (59.1) |
| **Race, n (%)** |  |  |  |
| American Indian or Alaska Native | 3 (3.3) | 0 | 3 (1.6) |
| Asian | 2 (2.2) | 1 (1.0) | 3 (1.6) |
| Black or African American | 22 (24.4) | 23 (24.0) | 45 (24.2) |
| Multiple | 2 (2.2) | 1 (1.0) | 3 (1.6) |
| White | 61 (67.8) | 71 (74.0) | 132 (71.0) |
| **Ethnicity, n (%)** |  |  |  |
| Hispanic or Latino | 21 (23.3) | 28 (29.2) | 49 (26.3) |
| Not Hispanic or Latino | 69 (76.7) | 68 (70.8) | 137 (73.7) |
| **HbA1c, %** | 6.03 ± 0.24 | 6.09 ± 0.21 | 6.06 ± 0.23 |
| **Fasting serum glucose, mg/dL** | 103.2 ± 11.41 | 101.9 ± 11.98 | 102.5 ± 11.70 |
| **Fasting insulin, pmol/L** | 160.6 ± 83.18 | 175.9 ± 93.05 | 168.5 ± 88.51 |
| **Duration of obesity, years** | 18.0 ± 12.61 | 17.4 ± 13.10 | 17.7 ± 12.83 |
| **Body Weight, kg** | 117.4 ± 26.47 | 120.3 ± 31.99 | 118.9 ± 29.40 |
| **BMI, kg/m^2^** | 40.6 ± 8.275 | 41.8 ± 9.62 | 41.2 ± 8.99 |
| <35 kg/m^2^, n (%) | 22 (24.4) | 22 (22.9) | 44 (23.7) |
| ≥35 kg/m^2^, n (%) | 68 (75.6) | 74 (77.1) | 142 (76.3) |
| **Waist circumference, cm** | 120.8 ± 15.81 | 124.4 ± 20.18 | 122.7 ± 18.24 |
| **Waist-to-height ratio** | 0.71 ± 0.08 | 0.73 ± 0.11 | 0.72 ± 0.10 |
| **Systolic blood pressure, mmHg** | 126.8 ± 12.56 | 130.1 ± 12.52 | 128.5 ± 12.62 |
| **Diastolic blood pressure, mmHg** | 81.2 ± 8.01 | 81.5 ± 8.36 | 81.3 ± 8.17 |
| **Pulse rate, bpm** | 72.5 ± 9.17 | 71.3 ± 9.53 | 71.9 ± 9.35 |
| **eGFR, mL/min/1.73m^2^** | 101.0 ± 17.77 | 101.7 ± 18.23 | 101.4 ± 17.96 |
| **Hypertension, n (%)** | 46 (51.1) | 49 (51.0) | 95 (51.1) |
| **Dyslipidemia, n (%)** | 36 (40.0) | 38 (39.6) | 74 (39.8) |
| **Number of ORCs, including prediabetes, n (%)** |  |  |  |
| 1 | 17 (18.9) | 21 (21.9) | 38 (20.4) |
| 2 | 28 (31.1) | 32 (33.3) | 60 (32.3) |
| ≥3 | 45 (50.0) | 43 (44.8) | 88 (47.3) |

Data are mean ± SD, unless otherwise indicated, in participants with prediabetes at randomization from the modified intent-to-treat population. Abbreviations: BMI=body mass index; eGFR=estimated glomerular filtration rate (from creatinine adjusted for body surface area); HbA1c=glycated hemoglobin; MTD=maximum tolerated dose; ORCs=obesity-related complications; SD=standard deviation.

**Table S2. Body weight reduction-associated and -unassociated changes on HOMA2-IR (computed with insulin) in participants with prediabetes from SURMOUNT-5**

| **HOMA2-IR** | **Total effect**  **(change from baseline at Week 72**  **tirzepatide MTD vs semaglutide MTD)** | **Weight-loss independent attribution (%)** | **Weight-loss dependent attribution (%)** |
| --- | --- | --- | --- |
| **Study-defined Prediabetes** |  |  |  |
| **HOMA2-IR (computed with insulin)** | -0.27 (-0.39, -0.16) | 48.3 | 51.7 |
| **WHO/IEC-defined Prediabetes** |  |  |  |
| **HOMA2-IR (computed with insulin)** | -0.29 (-0.44, -0.13) | 61.6 | 38.4 |

Change from baseline at Week 72 are presented as estimate using log transformation. Abbreviations: HOMA2-IR=homeostasis model assessment of insulin resistance; MTD=maximum tolerated dose.

**Table S3. Safety profile**

|  | **Overall Population** | | | **Study-defined Prediabetes** | | |
| --- | --- | --- | --- | --- | --- | --- |
| **Parameter** | **Tirzepatide MTD**  **(10 or 15 mg)**  **N=374** | **Semaglutide MTD**  **(1.7 or 2.4 mg)**  **N=376** | **Total**  **Population**  **N=750** | **Tirzepatide MTD**  **(10 or 15 mg)**  **N=215** | **Semaglutide MTD**  **(1.7 or 2.4 mg)**  **N=210** | **Total Population**  **N=425** |
| **Participants with ≥1 TEAE** | 287 (76.7) | 297 (79.0) | 584 (77.9) | 170 (79.1) | 175 (83.3) | 345 (81.2) |
| **Adverse events occurring in ≥5% of participants^a^** |  |  |  |  |  |  |
| Nausea | 163 (43.6) | 167 (44.4) | 330 (44.0) | 98 (45.6) | 92 (43.8) | 190 (44.7) |
| Constipation | 101 (27.0) | 107 (28.5) | 208 (27.7) | 59 (27.4) | 65 (31.0) | 124 (29.2) |
| Diarrhea | 88 (23.5) | 88 (23.4) | 176 (23.5) | 53 (24.7) | 52 (24.8) | 105 (24.7) |
| Vomiting | 56 (15.0) | 80 (21.3) | 136 (18.1) | 32 (14.9) | 45 (21.4) | 77 (18.1) |
| COVID-19 | 51 (13.6) | 47 (12.5) | 98 (13.1) | 30 (14.0) | 28 (13.3) | 58 (13.6) |
| Fatigue | 39 (10.4) | 46 (12.2) | 85 (11.3) | 24 (11.2) | 28 (13.3) | 52 (12.2) |
| Eructation | 37 (9.9) | 29 (7.7) | 66 (8.8) | 20 (9.3) | 16 (7.6) | 36 (8.5) |
| Upper respiratory tract infection | 32 (8.6) | 43 (11.4) | 75 (10.0) | 19 (8.8) | 28 (13.3) | 47 (11.1) |
| Injection site reaction | 32 (8.6) | 1 (0.3) | 33 (4.4) | 18 (8.4) | 0 | 18 (4.2) |
| Alopecia | 31 (8.3) | 23 (6.1) | 54 (7.2) | 16 (7.4) | 13 (6.2) | 29 (6.8) |
| Abdominal distension | 27 (7.2) | 24 (6.4) | 51 (6.8) | 14 (6.5) | 15 (7.1) | 29 (6.8) |
| Dizziness | 24 (6.4) | 18 (4.8) | 42 (5.6) | 14 (6.5) | 12 (5.7) | 26 (6.1) |
| Gastroesophageal reflux disease | 23 (6.1) | 40 (10.6) | 63 (8.4) | 14 (6.5) | 26 (12.4) | 40 (9.4) |
| Abdominal pain | 24 (6.4) | 26 (6.9) | 50 (6.7) | 13 (6.0) | 13 (6.2) | 26 (6.1) |
| Flatulence | 18 (4.8) | 8 (2.1) | 26 (3.5) | 13 (6.0) | 4 (1.9) | 17 (4.0) |
| Headache | 27 (7.2) | 27 (7.2) | 54 (7.2) | 13 (6.0) | 13 (6.2) | 26 (6.1) |
| Influenza | 14 (3.7) | 13 (3.5) | 27 (3.6) | 11 (5.1) | 10 (4.8) | 21 (4.9) |
| Decrease appetite | 17 (4.5) | 19 (5.1) | 36 (4.8) | 10 (4.7) | 14 (6.7) | 24 (5.6) |
| Dyspepsia | 22 (5.9) | 28 (7.4) | 50 (6.7) | 9 (4.2) | 17 (8.1) | 26 (6.1) |
| Nasopharyngitis | 17 (4.5) | 23 (6.1) | 40 (5.3) | 9 (4.2) | 15 (7.1) | 24 (5.6) |
| Sinusitis | 11 (2.9) | 21 (5.6) | 32 (4.3) | 7 (3.3) | 13 (6.2) | 20 (4.7) |
| Back pain | 7 (1.9) | 16 (4.3) | 23 (3.1) | 5 (2.3) | 11 (5.2) | 16 (3.8) |

Data are n (%) from the safety population.

^a^ Adverse events occurring in ≥5% of participants treated with tirzepatide or semaglutide from either subgroup are listed according to preferred terms in the Medical Dictionary for Regulatory Activities, version 27.1.

Abbreviations: COVID=19=coronavirus disease of 2019; MTD=maximum tolerated dose; TEAE=treatment-emergent adverse event.

**Figure S1. Change in additional** **HOMA2 parameters in participants with study-defined prediabetes from SURMOUNT-5**

Data are overall baseline geometric mean, estimate (SE) actual values and percent change from baseline at Week 72 using log transformation from the efficacy analysis set. **p<0.001 change from baseline; ##p<0.001 vs semaglutide. (A) HOMA2-S (computed with insulin) over time and percent change from baseline at Week 72. (B) HOMA2-B (computed with insulin) over time and percent from baseline in at Week 72. Abbreviations: HOMA2-B=homeostatic model assessment for beta-cell function; HOMA2-S=homeostatic model assessment for insulin sensitivity; MTD=maximum tolerated dose; SE=standard error.

**Figure S2. Change in glycemic parameters in participants with WHO/IEC-defined prediabetes from SURMOUNT-5**

Data are LSM (SE) actual values over time and change from baseline at Week 72 from the efficacy analysis set. For fasting insulin and HOMA2 parameters, values are overall baseline geometric mean, estimate (SE) actual values and percent change from baseline at Week 72 using log transformation. **p<0.001 change from baseline; #p<0.05 and ##p<0.001 vs semaglutide. (A) HbA1c (%) over time and change from baseline at Week 72. (B) Fasting serum glucose (mg/dL) over time and change from baseline at Week 72. (C) Fasting insulin (pmol/L) over time and change from baseline at Week 72. (D) HOMA2-IR (computed with insulin) over time and percent from baseline at Week 72. (E) HOMA2-S (computed with insulin) over time and percent change from baseline at Week 72. (F) HOMA2-B (computed with insulin) over time and percent change from baseline in at Week 72. Abbreviations: HOMA2-B=homeostatic model assessment for beta-cell function; HOMA2-IR=homeostatic model assessment for insulin resistance; HOMA2-S=homeostatic model assessment for insulin sensitivity; LSM=least squares mean; MTD=maximum tolerated dose; SE=standard error.

**Figure S3. Change in glycemic parameters in participants with WHO/IEC-defined prediabetes from SURMOUNT-5**

Data are percentage of participants based on imputed data from the efficacy analysis set. #p<0.05 and ##p<0.001 vs semaglutide. Note: Missing data at Week 72 due to early treatment discontinuation, initiation of prohibited medications or bariatric surgery or other weight loss procedures were assumed to be missing at random. In this case, multiple imputation was based on data from participants in the same treatment arm. Missing data were imputed 100 times. Statistical inference for endpoint uses Rubin's Rule to combine analyses of imputed datasets. (A) Proportion of participants with WHO/IEC-defined prediabetes achieving HbA1c targets at Week 72. (B) Proportion of participants with WHO/IEC-defined prediabetes achieving body weight reduction thresholds at Week 72. Abbreviations: MTD=maximum tolerated dose.

**Figure S4. Change in body weight-related parameters in participants with WHO/IEC-defined prediabetes from SURMOUNT-5**

Data are LSM (SE) actual values and change or percent change from baseline at Week 72 from the efficacy analysis set. **p<0.001 change from baseline; ##p<0.001 vs semaglutide. (A) Body weight (kg) over time and percent change from baseline at Week 72 in participants with WHO/IEC-defined prediabetes. (B) Waist circumference (cm) over time and change from baseline at Week 72 in participants with WHO/IEC-defined prediabetes. (C) Waist-to-height ratio over time and change from baseline at Week 72 in participants with WHO/IEC-defined prediabetes. Abbreviations: LSM=least squares mean; MTD=maximum tolerated dose; SE=standard error.

**Figure S5. Relationship between changes in body weight (kg) and HOMA2 parameters at Week 72 in participants with study-defined prediabetes from SURMOUNT-5**

Data are change in body weight (kg) and change in HOMA2 parameters from baseline to Week 72 from the efficacy analysis set. Abbreviations: HOMA2=Homeostatic Model Assessment; SEMA=semaglutide; TZP=tirzepatide.

**Figure S6. Relationship between changes in body weight (kg) and HOMA2 parameters at Week 72 in participants with WHO/IEC-defined prediabetes from SURMOUNT-5**

Data are change in body weight (kg) and change in HOMA2 parameters from baseline to Week 72 from the efficacy analysis set. Abbreviations: HOMA2=Homeostatic Model Assessment; SEMA=semaglutide; TZP=tirzepatide.
